# SARS-CoV-2 receptor binding domain-specific antibodies activate platelets with features resembling the pathogenic antibodies in heparin-induced thrombocytopenia

**DOI:** 10.21203/rs.3.rs-462080/v1

**Published:** 2021-04-26

**Authors:** Wen Zhu, Yongwei Zheng, Mei Yu, Jianhui Wei, Yongguang Zhang, Paytsar Topchyan, Christine Nguyen, Rae Janecke, Lisa Baumann Kreuziger, Gilbert C. White, Parameswaran Hari, Richard Aster, Weiguo Cui, Shawn Jobe, Mary Beth Graham, Demin Wang, Renren Wen

**Affiliations:** Blood Research Center; Blood Research Center; Blood Research Center; Fujian Normal University; Fujian Normal University; Blood Research Center; Blood Research Center; Blood Research Center; Blood Research Center; Blood Research Center; Medical College of Wisconsin; Blood Research Center; Blood Research Center; Blood Research Center; Blood Research Center; Blood Research Center; Blood Research Center

**Keywords:** heaprin induced thrombocytopenia, covid-19, platelet-activating antibodies

## Abstract

Severe COVID-19 is associated with unprecedented thromboembolic complications. We found that hospitalized COVID-19 patients develop immunoglobulin Gs (IgGs) that recognize a complex consisting of platelet factor 4 and heparin similar to those developed in heparin-induced thrombocytopenia and thrombosis (HIT), however, independent of heparin exposure. These antibodies activate platelets in the presence of TLR9 stimuli, stimuli that are prominent in COVID-19. Strikingly, 4 out of 42 antibodies cloned from IgG1^+^ RBD-binding B cells could activate platelets. These antibodies possessed, in the heavy-chain complementarity-determining region 3, an RKH or Y_5_ motif that we recently described among platelet-activating antibodies cloned from HIT patients. RKH and Y_5_ motifs were prevalent among published RBD-specific antibodies, and 3 out of 6 such antibodies tested could activate platelets. Features of platelet activation by these antibodies resemble those by pathogenic HIT antibodies. B cells with an RKH or Y_5_ motif were robustly expanded in COVID-19 patients. Our study demonstrates that SARS-CoV-2 infection drives the development of a subset of RBD-specific antibodies that can activate platelets and have activation properties and structural features similar to those of the pathogenic HIT antibodies.

## Introduction

The majority of people infected with severe acute respiratory syndrome coronavirus 2 (SARS-CoV-2) only develop mild symptoms, whereas a small subset of patients develop severe coronavirus disease 2019 (COVID-19) and some succumb to the disease^1^. Severe COVID-19 is a multisystemic disease, and thromboinflammation is emerging as a major cause of morbidity and mortality^2^. Clinical coagulopathy associated with severe COVID-19 is characterized by significant elevation of plasma D-dimer, moderate prolongation of prothrombin time and partial thromboplastin time, and mild thrombocytopenia^3–7^. Thromboinflammation in severe COVID-19 is characterized by increased plasma inflammatory markers, such as IL-6, tumor necrosis factor alpha, C-reactive protein, and activation of complement pathway^3^, and dysregulated activation of cellular components participating in inflammatory and coagulation responses that include platelets^8^, endothelium^9^, monocytes^10^, and neutrophils^11^. Thrombotic manifestations range from arterial thrombosis, venous thromboembolism, to tissue micro thrombosis^12^ and occur in at least 20% of COVID-19 patients in intensive care unit (ICU), but perhaps more based on postmortem studies^13^. Of note, clinical manifestations, frequency, plasma biomarkers, and hematological changes seen in SARS-CoV-2-infected patients resemble those typical of heparin-induced thrombocytopenia and thrombosis (HIT)^14^.

HIT is an antibody-mediated reaction in patients exposed to heparin, characterized by *thrombocytopenia and venous and arterial thrombosis^15^.* HIT is caused by formation of immunoglobulin Gs (IgGs), primarily IgG1s^16^, that recognize a complex formed between a platelet a-granule protein platelet factor 4 and heparin (PF4/H) or PF4/polyanion. In HIT, platelet-activating PF4/H-reactive antibodies drive a prothrombotic state through activation of Fc receptors on platelets^17^, monocytes^18^, and neutrophils^19^ and through activation of an unidentified molecule on endothelial cells^20^. “Spontaneous” HIT can arise in the absence of proximate heparin exposure and is similarly initiated by the formation of anti-PF4/H platelet-activating antibodies^21^.

We recently cloned from HIT patients a group of PF4/H-binding IgG1 antibodies that included platelet-activating clones^22,23^. PF4/H-reactive clones had longer heavy-chain complementarity determining region (HCDR3). Platelet-activating clones contained either an RX_1–2_R/K/HX_1–2_R/K/H or a string of 5 tyrosine residues in an unusually long HCDR3 (≥ 20 amino acid residues), which we named RKH or Y_5_ motif respectively^22–23^. Interestingly, a group of antibodies reactive to the receptor-binding domain (RBD) of SARS-CoV-2 spike protein have longer HCDR3^24^. Strikingly, clones possessing an RKH or Y_5_ motif have been identified among RBD-binding clones or as dominant clones in severe COVID-19 patients^24–25^. In the current study, we investigate whether severe COVID-19 patients develop platelet-activating and prothrombotic antibodies similar to those that drive the pathologic manifestations of HIT, however, independent of heparin exposure and whether RBD-specific antibodies can activate platelets with features similar to those cloned from HIT patients.

## Results

### COVID-19 patients enrolled in the study

Plasma and peripheral blood samples were collected from COVID-19 patients enrolled in “An Open label, Phase 2 Study Evaluating the Efficacy and Safety of High-Titer Anti-SARS-CoV2 plasma in hospitalized patients with COVID-19 infection” (NCT04354831). Patient information is presented in Table S1. Samples collected from 40 patients on day 0 of enrollment were analyzed. Plasma samples from 10 HIT patients (diagnosed based on clinical manifestation, positive PF4-reactive antibody assay, and positive serotonin release assay) and 8 deidentified healthy individuals were used as controls. The studies were approved by the Institutional Review Boards of the Medical College of Wisconsin and the Froedtert Hospital.

### Hospitalized COVID-19 patients developed PF4/H-reactive and platelet-activating antibodies that are independent of heparin exposure

**Figure 1:**
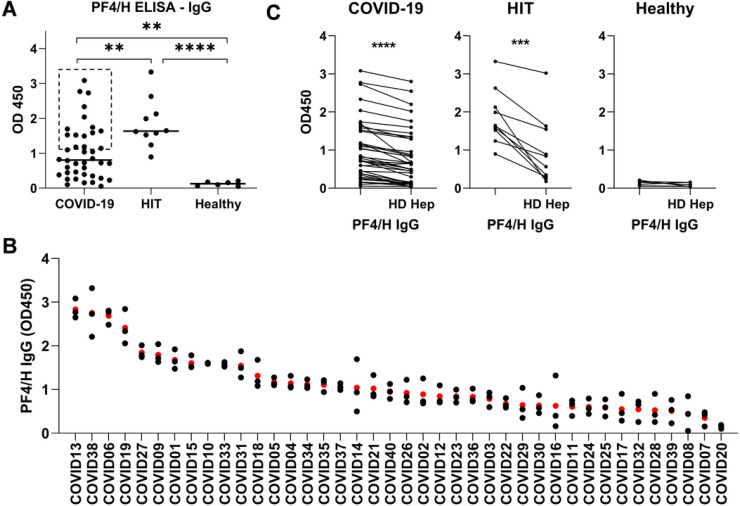
Hospitalized COVID-19 patients develop PF4/H-reactive IgG antibodies. A. PF4/H-reactive IgG antibodies were increased in COVID-19 patients compared to healthy controls. B. A plot of 3 independent measurements of PF4/H-reactive IgG level in COVID-19 patient plasma. The average level of PF4/H-reactive IgG for each patient was presented by the red dots. C. Binding of IgG antibodies to PF4/H was inhibited by high-dose (HD) heparin. Plasma samples from 40 hospitalized COVID-19 patients were tested. 100 x diluted plasma was used in the assays. Data shown in A and C were representative of 3 independent experiments. P-values were calculated by unpaired (A) and paired (C) two-tailed Student’s t-test. *: p<0.05, **: p<0.01, ***: p<0.001, ****: p<0.0001. The same symbols representing the corresponding p-values were used throughout the paper unless stated otherwise.

We investigated whether PF4/H-reactive antibodies are elicited in the setting of SARS-CoV-2 infection using the enzyme linked immunosorbent assay (ELISA) for PF4/H-reactive antibodies in hospitalized COVID-19 patients. Levels of plasma PF4/H-reactive IgG and IgM were both markedly higher in hospitalized COVID-19 patients than in healthy controls (Figs. 1A, 1B, and S1A), and PF4/H-reactive IgG level of the top 43% COVID-19 samples was similar to that in patients with HIT (Fig. 1A, data in dashed rectangle). Excess heparin inhibited binding of plasma IgG and IgM antibodies in the COVID-19 patients, demonstrating the similarity of SARS-CoV-2 induced PF4/H-reactive antibodies to those elicited in the context of HIT (Figs. 1C and S1B).

**Figure 2:**
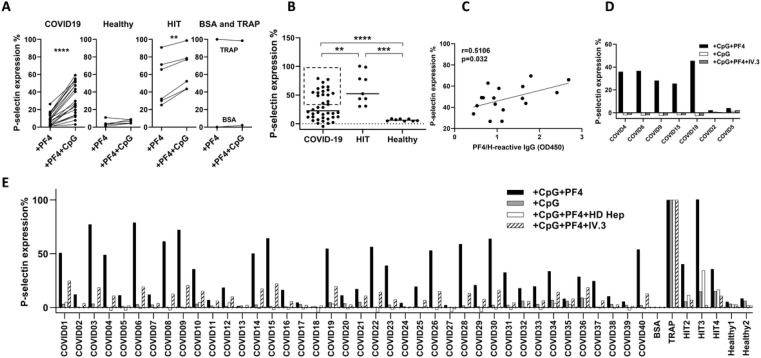
Hospitalized COVID-19 patients have HIT-like platelet-activating antibodies. A. CpG treatment significantly enhanced platelet activation by COVID-19 and HIT plasma, but not healthy plasma in PEA. TRAP (thrombin receptor activating peptide) was used as a positive control. B. Plasma from COVID-19 patients could activate platelets in the PEA+CpG. C. The top 40% PEA+CpG-positive samples from COVID-19 patients were correlated with the level of PF4/H-reactive IgGs. D. Purified IgG antibodies from PEA+CpG-positive but not PEA+CpG-negative COVID-19 plasma samples were able to activate platelets in the PEA+CpG. E. Platelet activation by COVID-19 plasma was PF4 and FcγRIIA-dependent and inhibited by HD Heparin. Plasma samples from 40 hospitalized COVID-19 patients (COVID1–40) were tested by the PEA+CpG in the presence or absence of exogenously added PF4, or in the presence of IV.3 or HD heparin. Plasma samples from HIT patients (HIT2–4) and healthy people (Healthy 1 and 2) were included as controls. Figs. A-C and E represent 3 independent experiments and Fig. D is a representative of 2 independent experiment.

To examine whether platelet-activating antibodies are induced in the context of SARS-CoV-2 infection, we used the PF4-dependent P-selectin expression assay (PEA)^26^. In this assay, the ability of patient plasma to induce platelet activation is tested in the context of added PF4, and platelet activation is measured by the externalization of P-selectin. Plasma from COVID-19 inpatients induced higher levels of P-selectin expression on platelets compared to healthy controls (Fig. 2A). Since COVID-19 patient plasma has elevated levels of neutrophil extracellular traps (NETs) that are prominent in COVID-19 and are known to activate TLR9^27,28^, we investigated whether TLR9 agonist CpG could co-stimulate platelets and thus enhance platelet responsiveness to platelet-activating antibodies in the PEA. Indeed, CpG substantially enhanced platelet responsiveness to platelet-activating antibodies cloned from HIT patients but had no effect on platelet activation when non-activating clones were added (Fig. S2). CpG co-stimulation in PEA (PEA^+CpG^) also markedly augmented platelet activation stimulated by plasma from COVID-19 patients, and this co-stimulatory effect of CpG was similarly observed in the context of HIT plasma (Fig. 2A). No platelet activation was induced by CpG in the context of plasma from healthy controls (Fig. 2A). We thus used PEA^+CpG^ to measure platelet activation induced by plasma from 40 COVID-19 patients and found that markedly higher levels of P-selectin was induced by patient plasma than healthy controls (Fig. 2B and S3A). Platelet activation induced by the top 43% plasma samples mirrored those of plasma from HIT patients (Fig. 2B, data in dashed rectangle). PF4/H-reactive IgGs and P-selectin induction by all the COVID-19 plasma tested were positively correlated (Fig. S2B), and this correlation was intensified among the top 43% of COVID-19 plasma samples with stronger platelet-activating abilities (Fig. 2C), similar to HIT antibodies. IgG was purified from COVID-19 patient plasma with and without reactivity in the PEA^+CpG^ assay. Only purified IgG from the PEA^+CpG^-positive plasma but not the PEA^+CpG^-negative plasma was able to activate platelets (Figs 2D and S3C). The activation was PF4-dependent and inhibited by IV.3 (a monoclonal FcgRIIA-blocking antibody), demonstrating the importance of PF4 and FcgRIIA in platelet-activation (Fig. 2D), similar to HIT antibodies. PF4 and FcgRIIA dependent platelet activation was also demonstrated in COVID-19 plasma (Fig. 2E). The similarity of SARS-CoV-2 induced platelet-activating antibodies to those developed in HIT was further demonstrated by the inhibition of antibody-induced platelet activation by excess heparin (Fig. 2E). Taken together, these data demonstrate that SARS-CoV-2 infection induces the production of platelet-activating antibodies with similar properties to antibodies produced in HIT.

**Figure 3:**
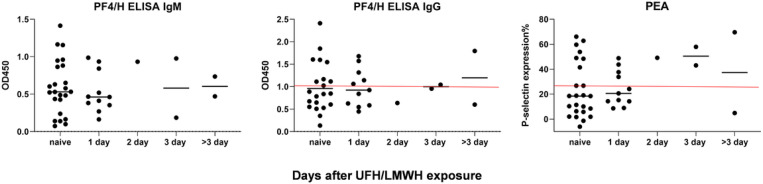
Hospitalized COVID-19 patients develop PF4/H-reactive and platelet-activating IgGs independent of UFH/LMWH exposure. Heparin-naïve refers to that patients were not exposed to UFH/LMWH at the time of blood collection for antibody measurement. The red lines represent the positive cutoff lines of the level of PF4/H-reactive IgGs or platelet-activating IgGs.

Since a large number of hospitalized COVID-19 patients are exposed to unfractionated heparin (UFH) or low molecular weight heparin (LMWH) either prophylactically or therapeutically, development of PF4/H-reactive and platelet-activating antibodies in the COVID-19 patients in our study could be attributed to heparin exposure. We thus searched UFH/LMWH exposure history of these patients (Table S1). We found that 83% (14 patients) of patients tested positive for PF4/H-reactive or platelet-activating IgGs (17 patients) (Fig. 3) were exposed to UFH/LMWH only 1–2 days (30%) or were heparin-naïve (53%) before blood collection. Since PF4/H-reactive antibodies become marginally detectable at least 3 days after UFH/LMWH exposure^29,30^, the development of PF4/H-reactive platelet-activating antibodies in hospitalized COVID-19 patients in the current study cannot be attributed to heparin.

### Some RBD-binding antibodies can activate platelets

**Figure 4:**
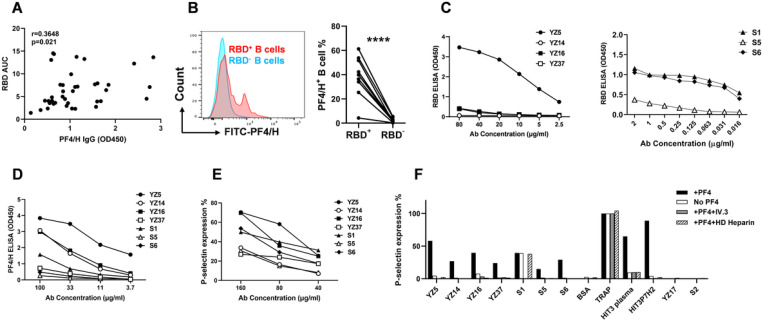
Some RBD-specific antibodies are platelet-activating antibodies. A. Levels of PF4/H-reactive and RBD-reactive IgGs in the plasma of COVID-19 patients were significantly correlated with each other. B. A large proportion of RBD-binding B cells also recognized PF4/H. C. RBD-binding curves of the 7 platelet-activating antibodies. D. PF4/H-binding curves of the 7 platelet-activating antibodies. E. PEA curves of the 7 platelet-activating antibodies. F. The activation properties of the seven platelet-activating antibodies. PEA was performed under the regular PEA condition, in the absence of exogenously added PF4, or in the presence of IV.3 or HD heparin. HIT3P7H2 is a platelet-activating antibody cloned from an HIT patient (HIT3). Data is a representative of 2–4 independent experiments.

Surprisingly, levels of PF4/H-reactive and RBD-reactive IgGs were positively correlated with each other (Figs. 4A and S4), suggesting potential cross-reactivity of a population of RBD-reactive IgGs with PF4/H. To investigate this possibility, we examined RBD and PF4/H cross-reactive B cells in the peripheral blood lymphocytes (PBMCs) of the COVID-19 patients by flow cytometry analysis. A high percentage of RBD-binding B cells also bound to PF4/H (39±16%) while PF4/H-binding was low in those B cells that did not bind RBD (2.2±1.8%) (Fig. 4B).

To investigate whether antibodies produced by the RBD-binding B cells activate platelets, we cloned antibodies from two patients, who had high-titer of RBD-specific antibodies and minimal heparin exposure. We sorted RBD^+^IgG1^+^ B cells (Fig. S5), and cloned 21 antibodies each from patient COVID39 (clones YZ1–17 and YZ39–42) and COVID40 (clones YZ18–38), respectively, using a single-cell PCR and expression cloning method. Among these, 4 platelet-activating clones were identified using PEA. Only a few of the antibodies cloned from the RBD-binding B cells bound to RBD, whereas 18 clones bound to PF4/H at a level comparable to those isolated from HIT patients^22^ (Figs. 4C and S6A). Of note, one (YZ5) of the 4 platelet-activating clones clearly bound to both PF4/H and RBD (Figs. 4C and 4D).

We further investigated whether higher affinity RBD-specific antibodies could activate platelets. Our recent studies in HIT have shown that PF4/H-binding and prothrombotic platelet-activating antibodies possess a k-chain with either an RKH or Y_5_ motif in their HCDR3^22,23^. Interestingly, a large number of RBD-specific antibodies possess an RKH or Y_5_ motif ^24,25^. Among antibodies cloned by Robbiani et al. from RBD-binding B cells in convalescent COVID-19 patients^25^, 15 possess an RKH or Y_5_ motif with some clonal expansion (Table S2 and data not shown). To investigate the platelet-activating potential of these high affinity RBD-specific clones, we expressed, based on the published sequences, 6 RKH or Y_5_ motif-possessing clones that also used a k-chain^25^ (Table S2). Three of these antibodies were platelet-activating clones (Clones S1, S5, S6). Compared to the platelet-activating antibodies cloned in this study from the RBD^+^IgG1^+^ B cells (YZ clones), clones S1, S5, and S6 bound much more strongly to RBD and induced equivalent platelet activation (Figs. 4C, 4E and S6B). Interestingly, S5 and S6 barely bound PF4/H (Fig. 4D). Thus, among the 7 platelet-activating antibodies identified from COVID-19 patients, 4 bound to RBD.

Of the 7 platelet-activating antibodies identified in the current study, activation by 6 antibodies was PF4 and FcgRIIA dependent and inhibited by high-dose heparin (Fig. 4F), indicating again that platelet activation by these antibodies function through a mechanism similar to that operates in HIT and also to that which has recently been described in vaccine-induced thrombocytopenia and thrombosis (VITT) following ChAdOx1n nCoV-19 and Ad26.COV2.S vaccination^31–33^. Different from other platelet-activating clones, clone S1 activated platelets in the absence of exogenously added PF4 and in the presence of high-dose heparin (Fig. 4F). In summary, a subset of RBD-specific antibodies induced by SARS-CoV-2 activate platelets and have functional characteristics resembling those of the prothrombotic and pathogenic antibodies of HIT and VITT.

### RKH and Y_5_ motifs are prominent in RBD-binding platelet-activating antibodies

**Figure 5:**
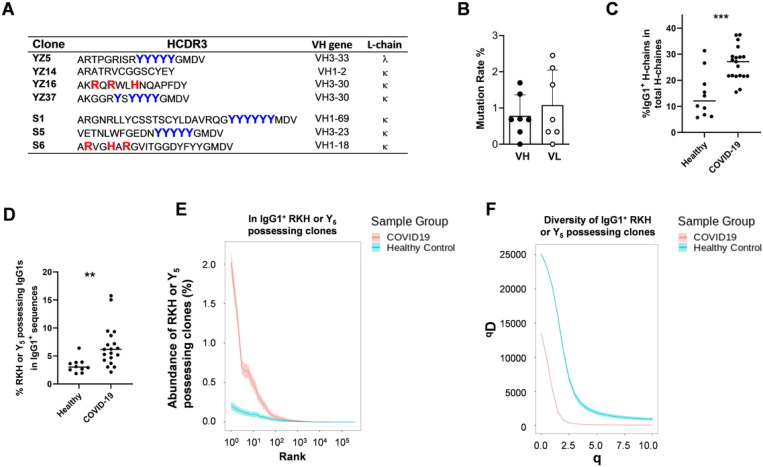
RKH and Y5 motifs are prominent in platelet-activating antibodies cloned from RBD-binding B cells in COVID-19 patients. A. The HCDR3 sequence of the platelet-activating antibodies cloned from RBD-binding B cells in COVID-19 patients. The RKH and Y5 motifs were shown in bold red or blue font respectively. B. The 7 platelet-activating antibodies have low mutation rate. C. IgG1+ B cells are significantly increase in COVID-19 patients compared to healthy controls. D. IgG1+ B cells that possess an RKH or Y5 motif are significantly more prevalent in COVID-19 patients compared to healthy controls. E. The abundance of the top ranked IgG1+ B cells that possess an RKH or Y5 motif are significantly higher in COVID-19 patients relative to healthy controls. The rank abundance distributions were calculated within the total reads of IgG1+ sequences that possessed an RKH or Y5 motif in the 19 COVID-19 patients and 10 healthy people with equivalent sampling size. F. Clonal diversity of the IgG1+ B cells that possess an RKH or Y5 motif are significantly reduced in COVID-19 patients relative to healthy controls. Clonal Diversity analysis was performed using the generalized diversity index proposed by Hill with uniform resampling from the total sequences possessing an RKH or Y5 motif from 19 COVID-19 patients or 10 healthy people to correct for sequencing depth.

Of the three RBD-specific platelet-activating antibodies identified based on their possession of the RKH or Y_5_ motifs, two possessed an Y_5_ motif (Fig. 5A, Clones S1 and S5) and one possessed an RKH motif (Fig. 5A, Clone S6). Strikingly, in the four platelet-activating antibodies cloned from RBD-binding B cells in our current study, clones YZ5 and YZ16 contained the Y_5_ and RKH sequences respectively and clone YZ37 contained the YXYYYY sequence that is very similar to the Y_5_ motif. Different from the platelet-activating antibodies identified in HIT, which contained the RKH or Y_5_ motif in an HCDR3 ≥ 20 amino acid residues, these three platelet-activating antibodies cloned in COVID-19 had a much shorter HCDR3^22,23^ (Fig. 5A). Thus, RKH or Y_5_ sequences appeared more important than the length of the HCDR3 in defining platelet-activating antibodies in COVID-19. In addition, whereas the 55 PF4/H-binding antibodies cloned from HIT patients exclusively used k-chains^22,23^, those cloned in COVID-19 patients, including one platelet-activating clone YZ5, also used l-chains (Fig. 5A). Hereafter, RX_1–2_R/K/H-X_1–2_R/K/H and YYYYY sequences will be called RKH and Y_5_ motifs, respectively, regardless of HCDR3 length. These data demonstrate that RBD-specific platelet-activating antibodies induced by SARS-CoV-2 infection bear structural motifs similar to those of platelet-activating antibodies induced in HIT.

Four of the 7 platelet-activating clones identified in the current study used VH3, including 2 which used VH3–30, and the remaining 3 used VH1, of which 1 used VH1–69 (Fig. 5A). VH3 and VH1 usage was also increased in the PF4/H-binding antibodies cloned from the RBD-binding B cells (Fig. S7). All of the platelet-activating clones and the majority of the PF4/H-binding clones displayed limited somatic mutation (Figs. 5B and S8). VH3 and VH1, in particular, VH3–30 and VH1–69 are over-represented in the spike-specific and RBD-specific antibodies induced in response to SARS-CoV-2 infection, and the majority of these antibodies have near germline sequences^24,25,34,35^. Thus, the structural signatures of the platelet-activating and PF4/H-binding clones that we have identified in COVID-19 patients are similarly enriched among the anti-spike and anti-RBD antibodies that have been previously reported.

### B cells possessing an RKH or Y_5_ motif are expanded robustly in COVID-19 patients

To investigate whether RKH or Y_5_ motif -possessing B cells are expanded in COVID-19 patients, we compared the frequency of B cells possessing an RKH or Y_5_ motif in COVID-19 patients and healthy people based on the published data. We focused on IgG1 isotype given its relevance to HIT pathogenicity^16^. We analyzed the data generated by Galson et al.^36^ and Briney et al.^37^, which performed deep or ultra-deep sequencing of B-cell receptor repertoires of 19 COVID-19 patients and 10 healthy people. We found that IgG1^+^ B cells were significantly increased in COVID-19 patients compared to healthy controls (Fig. 5C). VH sequences possessing an RKH or Y_5_ motif were present in IgG1 isotype in healthy controls (Fig. 5D, 3.3±1.3%), and were significantly increased in COVID-19 patients (Fig. 5D, 6.7±3.7%). The abundance (in percentage) of the top ranked clones that possessed an RKH or Y_5_ motif were significantly increased in IgG1^+^B cells in COVID-19 patients relative to healthy controls (Figs. 5E and S9). Clonal expansion will reduce the diversity of B-cell receptor repertoire, which can be revealed by diversity indexes, including clonal diversity 50 (D50), Shannon index, and Simpson’s index. We calculated D50, Shannon index, and Simpson’s index of B cells possessing an RKH or Y_5_ motif, and found that these indices of diversity were all significantly reduced in the RKH or Y_5_ possessing IgG1^+^ and IgG3^+^ B cells in COVID-19 patients relative to healthy controls (Figs. 5F and S10). Thus, in COVID-19 patients, there is a robust expansion of B cells possessing an RKH or Y_5_ motif.

## Discussion

Here we demonstrate that in COVID-19, the induction of SARS-CoV-2 RBD-specific antibodies is closely associated with the induction of PF4/H reactive platelet-activating antibodies. These PF4/H-reactive antibodies bear striking similarities to the platelet-activating antibodies that drive the thrombotic manifestations of HIT and VITT. Specifically, COVID-19 patients had a markedly increased level of PF4/H-reactive platelet-activating IgGs, and the level of such antibodies correlated with RBD-reactive IgG level. A significant proportion of RBD-binding B cells in COVID-19 also recognized PF4/H. Among the 7 platelet-activating antibodies cloned from COVID-19 patients, 4 bound to the SARS-CoV-2 RBD, 6 had platelet-activating characteristics similar to prothrombotic antibodies of HIT and VITT, and 6 possessed structural motifs, RKH or Y_5_, of the prothrombotic antibodies of HIT. Finally, we demonstrated clonal expansion of RKH or Y_5_ motif -possessing B cells in the repertoire of COVID-19 patients.

Given the timing of heparin exposure, PF4/H-reactive platelet-activating antibodies in the plasma of the significant majority of hospitalized COVID-19 patients could not have been induced by heparin. In addition, among the 7 platelet-activating RBD-specific clones identified in the current study, 3 were from patient 39 who did not receive heparin and 3 were from out patients. Thus, the majority of platelet-activating antibodies detected were not elicited by heparin. Several other studies also detected increased PF4/H-reactive antibodies in COVID-19 patients in the context of heparin treatment and patients are suspected of developing “classical” HIT, i.e. heparin exposure-induced HIT^38–42^. A recent randomized trial reported that therapeutic dose of UFH and LMWH did not improve organ dysfunction in critically ill patients with COVID-19^43^. Thus, the induction of prothrombotic platelet-activating antibodies by heparin and SARS-CoV-2 individually and cooperatively and their association with critical illness merits careful investigation.

Althaus et al. reported that IgG fractions from COVID-19 patients induced an FcgRIIA–dependent platelet-activation, specifically procoagulant apoptotic platelet formation, and such activity in the plasma appeared to contribute to disease severity^44,45^. However, it is not clear whether these antibodies are heparin-dependent or not. We found that the levels of platelet-activating antibodies in COVID-19 plasma relative to the healthy controls were significantly elevated but still lower than those in HIT patients. Co-stimulation of platelets with the TLR9 agonist CpG and plasma from hospitalized COVID-19 patients induced platelet activation to a level similar to that induced by HIT plasma. Such elevated but sub-threshold level of platelet-activating antibodies in severe COVID-19 could be sufficient to activate platelets and induce a prothrombotic state in the presence of strong inflammatory stimuli, such as TLR9 agonist NETs, in severe COVID-19 patients.

In addition to inflammatory signals, circulating plasma PF4 and PF4 released from activated platelets in the context of tissue damage during SARS-CoV-2 infection may also potentiate the prothrombotic functions of platelet-activating antibodies. In this context, platelet-activation by PF4/H-reactive antibodies in HIT appears to be largely dependent on neo-epitope formation when PF4 associates with cell surface glycosaminoglycan^46,47^. Some studies have shown an increase of circulating plasma PF4 in patients with severe-to-critical COVID-19^12^, but others have demonstrated a reduction of PF4 in patients with poor prognosis^48^. Activated platelets and platelet-secretory cargo, including PF4, are detected in the airways of patients with severe COVID-19^49^. The level of PF4 is also increased in bronchoalveolar lavage, peripheral blood, and serum of SARS-CoV-2-infected rhesus macaques^50^. Plasma PF4 is strongly associated platelet activation, and the high degree of platelet activation and thrombocytopenia associated with severe COVID-19, will affect plasma PF4 levels during disease progression. A better understanding of local and circulating concentrations of plasma factors, including TLR9 and PF4, that potentiate the platelet response should help delineate the prothrombotic role of such antibodies in COVID-19 patients.

Although platelet-activating antibodies cloned from COVID-19 patients bear structural and platelet-activating characteristics resembling the antibodies in HIT, there are some differences between the two groups of antibodies. 3 platelet-activating antibodies from COVID-19 patients barely bound PF4/H, which indicates that platelet-activation is not necessarily dependent on antibody recognition of PF4/H. The four platelet-activating antibodies cloned from RBD-binding B cells in COVID-19 patients (YZ clones) had a much shorter HCDR3 than those isolated from HIT patients, which have an HCDR3 ≥ 20 amino acid residues^22,23^. Thus, RKH or Y_5_ sequences appear more important than the length of the HCDR3 in defining platelet-activating antibodies in COVID-19. Whereas all 55 PF4/H-binding antibodies cloned from HIT patients exclusively used k-chains^22,23^, those cloned in COVID-19 patients, including one platelet-activating clone YZ5, also used l-chains. These structural differences of platelet-activating antibodies may be attributable to different B-cell stimulating antigen(s) in HIT and COVID-19 patients, or alternatively, may be due to limited number of clones identified in both diseases.

A large proportion of RBD-binding B cells cross-reacted with PF4/H (Fig. 1B). However, whereas about 40% of antibodies cloned from RBD binding B cells could interact with PF4/H, the majority bound RBD only very weakly. We observed that PF4/H-reactive antibodies are largely auto-reactive and poly-reactive^23^ (Zhu et. Al., unpublished study) and propose that innate B cells produce these auto-reactive platelet-activating antibodies and are controlled by intrinsic and extrinsic tolerance mechanisms as demonstrated in mice^51–53^. We postulate that even with very low affinity to RBD, these RBD and PF4/H cross-reactive B cells can be activated by RBD in the presence of high inflammatory signals provided by SARS-CoV-2 infection^54^. Whereas the antibodies cloned in our current study (YZ clones), including several platelet-activating clones, bound to RBD only very weakly, the three platelet-activating antibodies identified based on possession of an RKH or Y_5_ motif (S clones) bound to RBD with intermediate to high affinity. Thus, in COVID-19, a subset of B cells with variable affinity to the RBD of the SARS-CoV-2 spike protein are activated and generate antibodies capable of PF4-dependent platelet activation. Some of these PF4/H and RBD cross-reactive antibodies have functional and structural characteristic analogous to antibodies observed in HIT and would be similarly expected to drive local and systemic thrombosis and contribute to the thrombotic complications prominent in SARS-CoV-2 infection. Given the recent description of a HIT-like clinical syndrome VITT following ChAdOxI nCov-19 and Ad26.COV2.S vaccination^31–33^, our findings also provide a potential mechanistic explanation for the clinical observations. We posit that studies of the structural and functional characteristics of the antibodies and B cells generated in VITT may demonstrate similarities of the pathogenic antibodies of VITT with those that we identify here in the setting of COVID-19.

In summary, SARS-CoV-2 infection activates RBD-specific B cells to make HIT-like platelet-activating antibodies that are defined by the RKH and Y_5_ motif. These antibodies are capable of PF4-dependent platelet activation and may therefore contribute to the thrombotic complications seen in SARS-CoV-2 infection as they do in HIT.

## Supplementary Material

Supplement 1

Supplement 2
