# Supplementary material for: SARS-CoV-2 receptor binding domain-specific antibodies activate platelets with features resembling the pathogenic antibodies in heparin-induced thrombocytopenia
Source: Res Sq. 2021 Apr 26:rs.3.rs-462080. Preprint. [Version 1] doi: 10.21203/rs.3.rs-462080/v1 (PMC8132233; doi:10.21203/rs.3.rs-462080/v1)
Supplement: Supplement 1 [file 9aadb9ef32edfc3e291bc9ef.pdf]

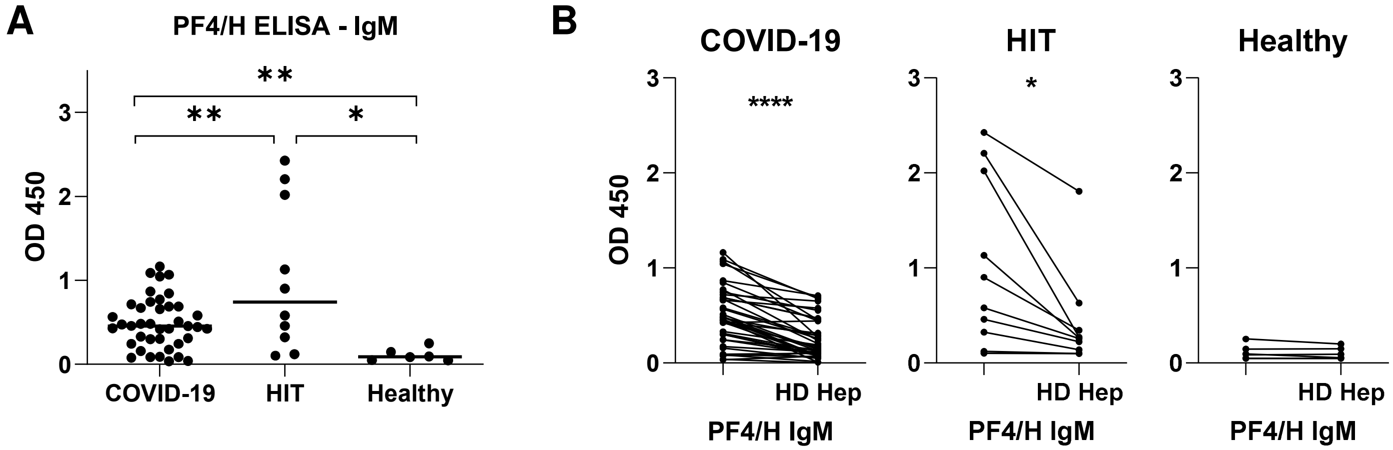

Fig. S1. Hospitalized COVID-19 patients develop PF4/H-reactive IgM antibodies. A. PF4/H-reactive IgM antibodies were increased in COVID-19 patients compared to healthy controls. **B.** Binding of IgM antibodies to PF4/H was inhibited by HD heparin. 100 x diluted plasma was used in the assays. Data shown were representative of 3 independent experiments. P-values were calculated by unpaired (A) and paired (B) two-tailed Student's t-test.

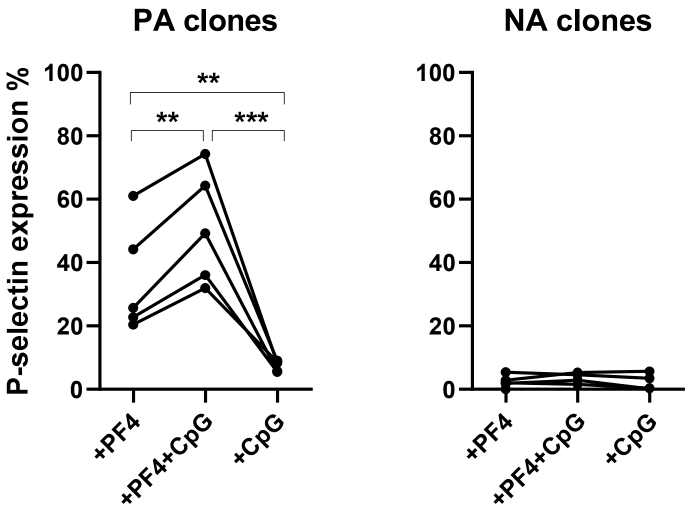

Fig. S2. CpG specifically increases platelet response to the platelet-activating antibody in PEA assay. Platelet-activating (PA) and non-activating (NA) clones were used in the PEA assay (+PF4) in the presence and absence of CpG. Platelets were also incubated with CpG alone (+CpG) as a control.

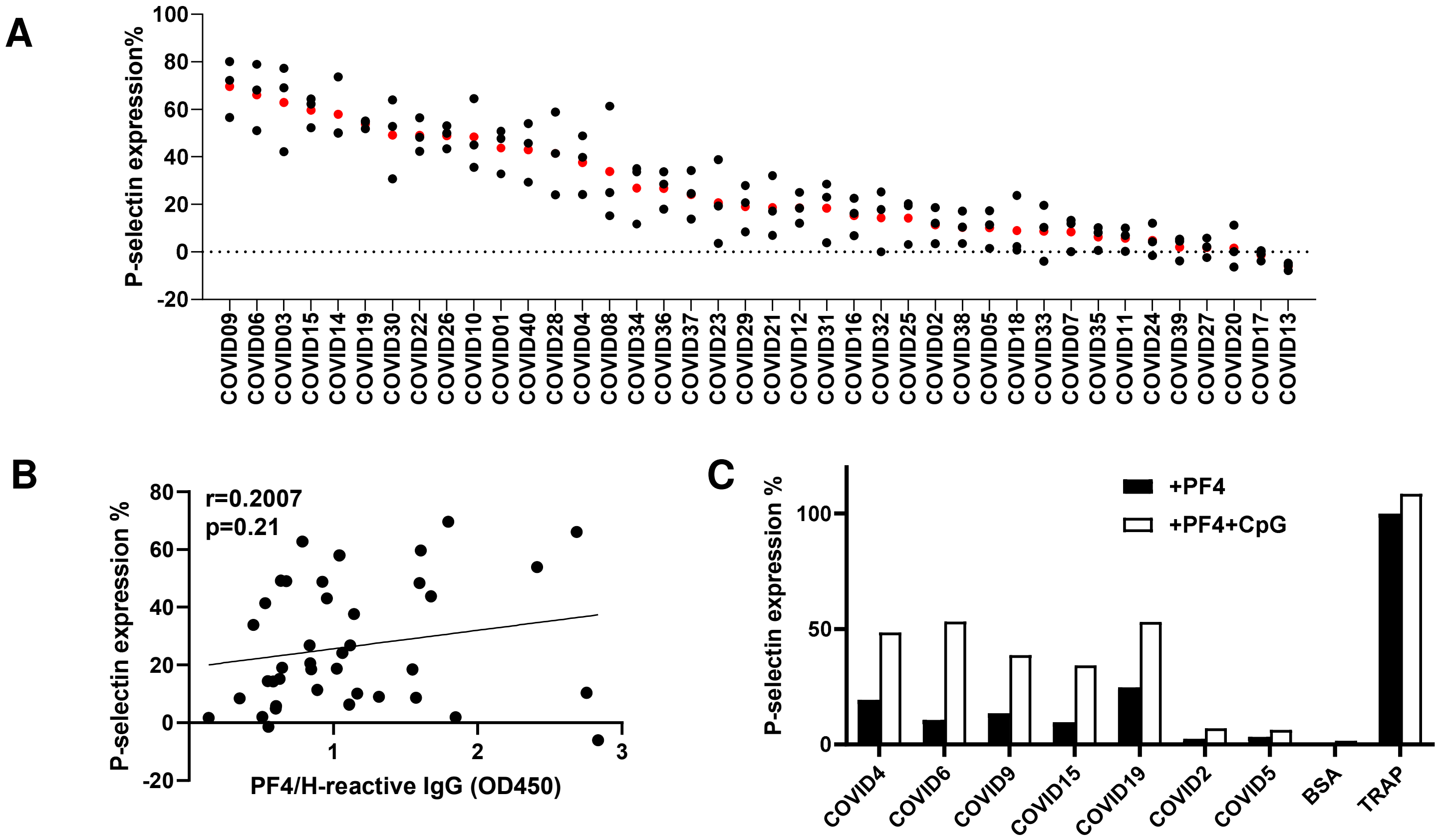

Fig. S3. Levels of platelet-activating antibodies in COVID-19 patients. A. A plot of 3 independent measurements of platelet activation (PEA^{+CpG}) induced by COVID-19 patient plasma. The average PEA^{+CpG} results for each patient was presented by the red dots. **B.** Level of PF4/H-reactive IgG is positively correlated with PEA^{+CpG} results. Correlation analysis was performed using the average results of three measurements of PF4/H-reactive IgG and PEA^{+CpG}. **C.** Platelet-activation induced by purified IgG (1.5 mg/ml) from COVID-19 patients in the PEA assay in the presence and absence of CpG (2 µg/ml).

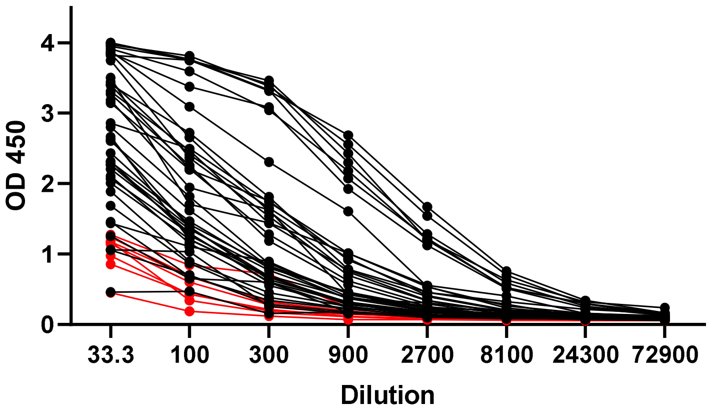

Fig. S4. RBD-specific IgG titers in the patient plasma.
Red lines represent healthy controls.

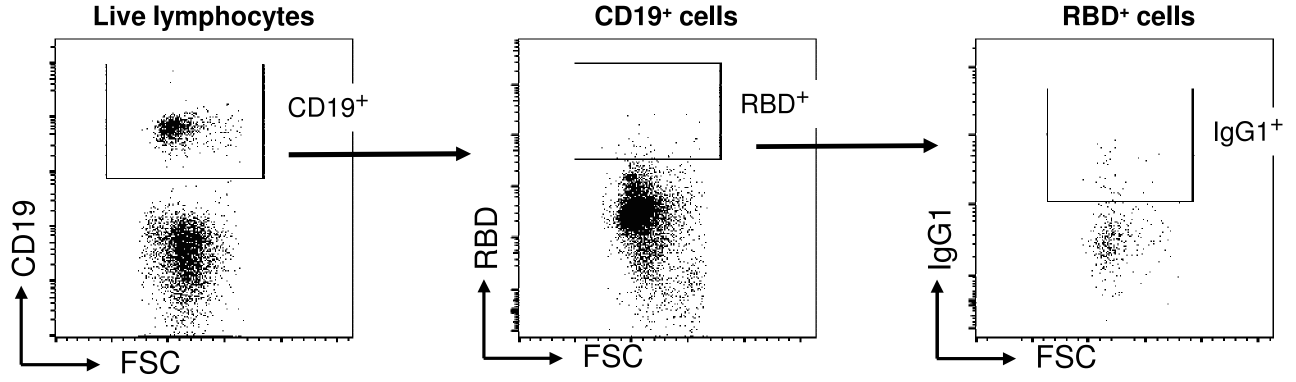

Fig. S5 Sorting strategy for IgG1⁺ RBD-binding B cells. PBMCs from COVID19-patients were isolated from citrated-blood and stained with the indicated antibodies to sort IgG1⁺ RBD⁺ B cells for antibody cloning.

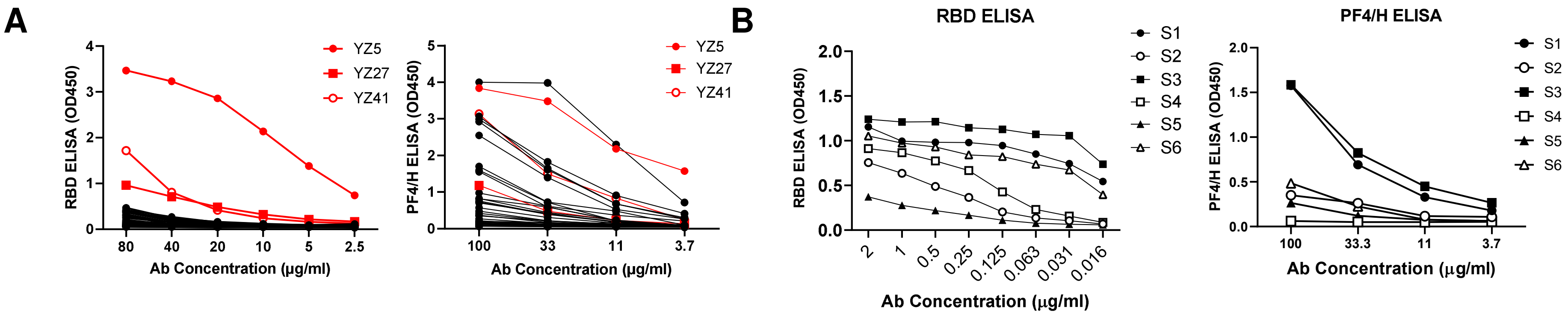

Fig. S6. Information on the antibodies cloned from RBD-binding B cells. A. RBD and PF4/H binding curves of the 42 YZ clones. Only RBD-binding clones with OD450≥1.0 (80 µg/ml) were presented by the red symbols in the RBD and PF4/H ELISA to differentiate them from the rest of the clones. **B.** RBD and PF4/H binding curves of the six S clones.

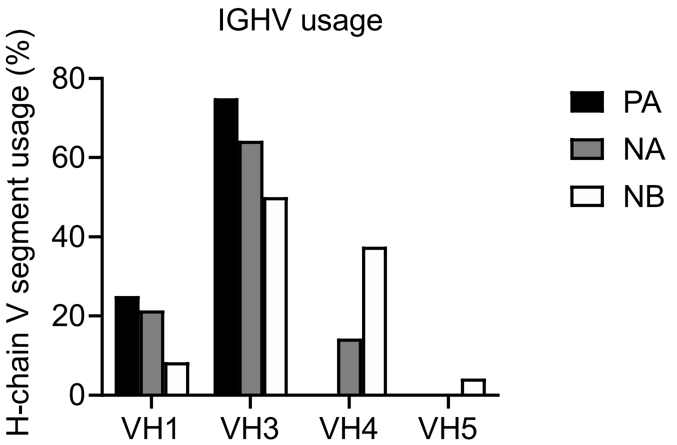

Fig. S7. VH1 and VH3 usage are increased in platelet-activating clones (PA) and PF4/H-binding but non platelet-activating clones (NA). NB=non PF4/H-binding clones. The analysis was performed on the 42 YZ clones, which included 4 PA clones, 14 NA clones, and 24 NB clones.

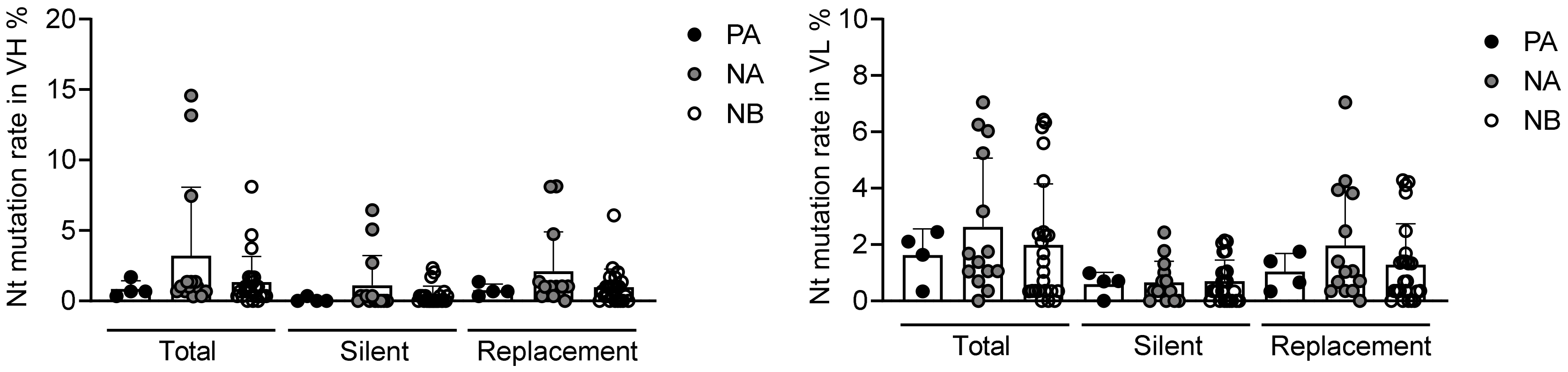

Fig. S8. Mutation rate in VH and VL of the PA, NA and NB clones. Mutation analysis of the 42 YZ clones, which included 4 PA clones, 14 NA clones, and 24 NB clones. PA= PF4/H-binding and platelet-activating clones, NA= PF4/H-bdining but non platelet-activating clones, NB=non PF4/H-binding clones, Nt= nucleotide

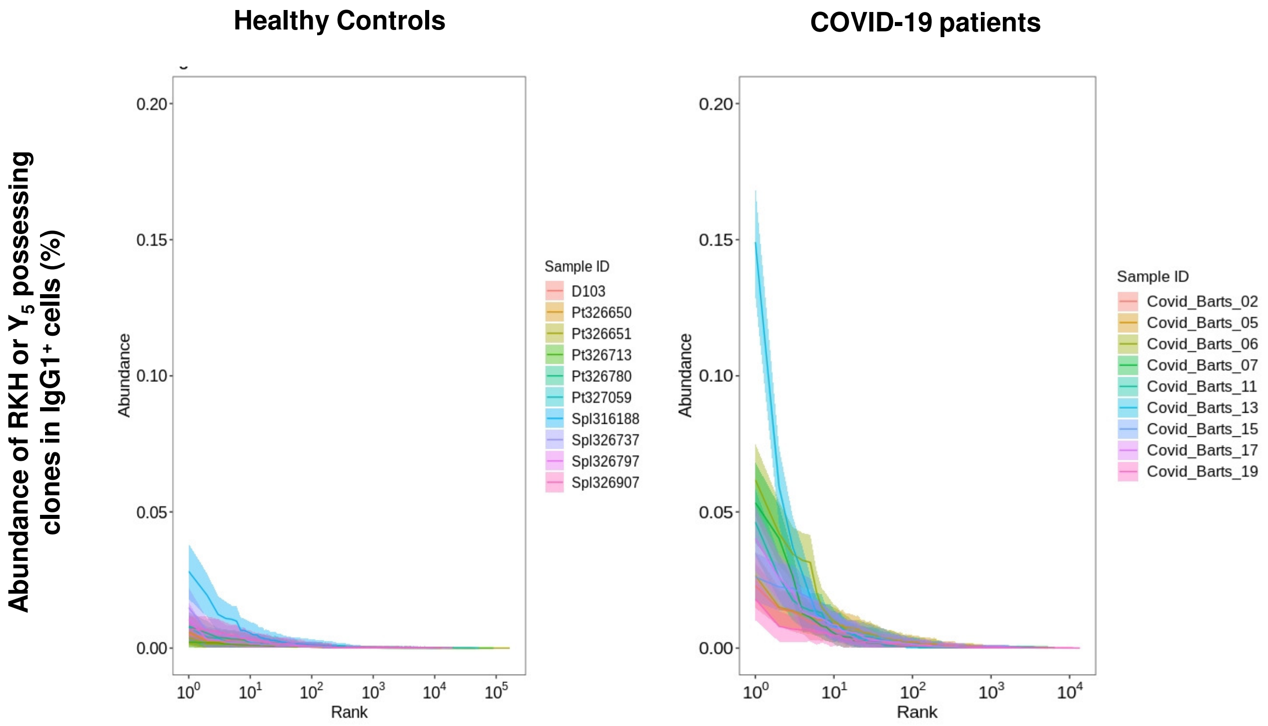

Fig. S9. Abundance of the ranked IgG1⁺ clones that possess an RKH or Y_5 motif is higher in COVID-19 patients compared to healthy controls. The rank abundance distributions represent all the IgG1⁺ sequences that possess an RKH or Y_5 motif sampled from COVID-19 patients and healthy people with resampling size of 1144 to correct for sequencing depth and samples that did not reach 1144 sequences were excluded from the analysis.

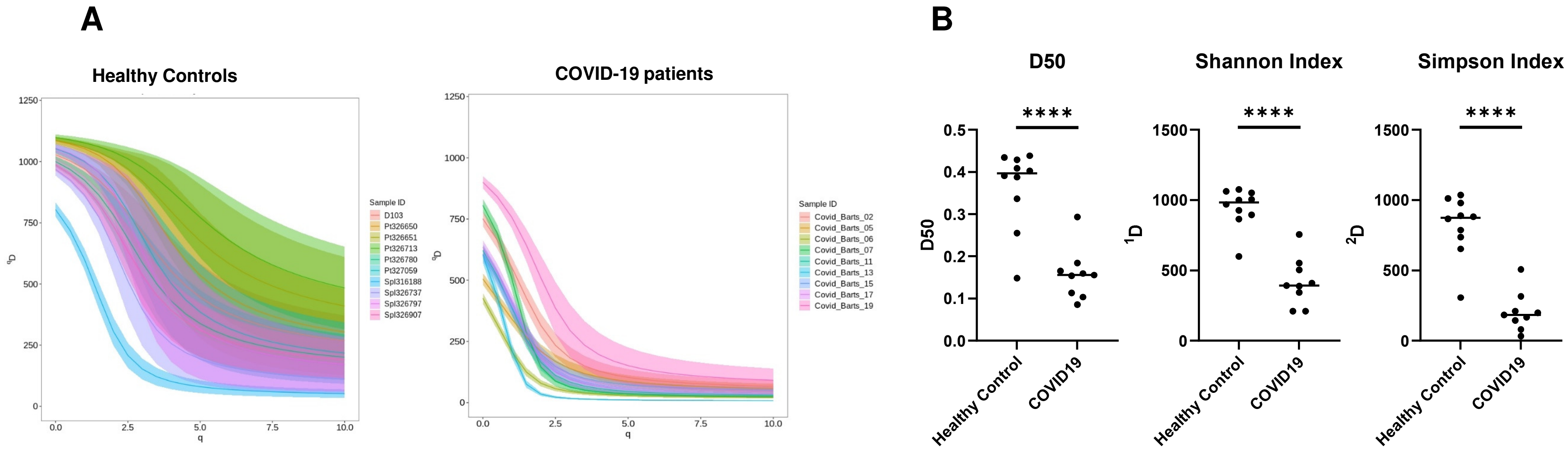

Fig. S10. Clonal diversity of IgG1⁺ B cells that possess an RKH or Y_5 motif is significantly reduced in COVID-19 patients compared to healthy controls. **A.** Diversity curves of IgG1⁺ B cells that possess an RKH or Y_5 motif in each subject with sampling size of 1144 per subject. **B.** Diversity 50 indexes (D50), Shannon diversity index (q=1), and Simpson diversity index (q=2) of IgG1⁺ B cells that possess an RKH or Y_5 motif in healthy subjects and COVID-19 patients. D50 was estimated based on the percentage of ranked clones that account for 50% of the total number of clones observed. Analysis was based on sampling size of 1144 per subject.
