# Supplementary material for: SARS-CoV-2 receptor binding domain-specific antibodies activate platelets with features resembling the pathogenic antibodies in heparin-induced thrombocytopenia
Source: Res Sq. 2021 Apr 26:rs.3.rs-462080. Preprint. [Version 1] doi: 10.21203/rs.3.rs-462080/v1 (PMC8132233; doi:10.21203/rs.3.rs-462080/v1)
Supplement: Supplement 2 [file ad74ed27df8d1c3ec1321db6.docx]

**Table S1. Patient information**

| **Patient ID** | **Age** | **Gender** | **Location^#^** | **D-dimer** | **Fibrinogen** | **Platelet count** | **Patient mortality at discharge** | **Days between enrollment and heparin/Enoxaparin exposure*** |
| --- | --- | --- | --- | --- | --- | --- | --- | --- |
| COVID01 | 57 | Female | Floor | 1.23 | 646 | 257 | Alive | 1 (Enoxaparin) |
| COVID02 | 76 | Male | ICU | 1.73 | 812 | 60 | Deceased | NA |
| COVID03 | 47 | Female | Floor | 0.34 | 506 | 188 | Alive | 0 (Enoxaparin) |
| COVID04 | 47 | Female | ICU | 0.41 | 462 | 276 | Alive | 1 (Enoxaparin) |
| COVID05 | 40 | Male | ICU | 0.92 | 620 | 130 | Alive | 0 (Enoxaparin) |
| COVID06 | 48 | Female | ICU | 0.49 | 860 | 234 | Deceased | 0 (Enoxaparin) |
| COVID07 | 53 | Male | Floor | 0.2 | 311 | 139 | Alive | 0 (Enoxaparin) |
| COVID08 | 86 | Male | Floor | 0.76 | 400 | 92 | Deceased | 1 (Enoxaparin) |
| COVID09 | 41 | Male | Floor | 0.48 | 549 | 261 | Alive | 8 (Enoxaparin) |
| COVID10 | 53 | Male | Floor | 0.55 | 773 | 187 | Alive | -3 (Enoxaparin) |
| COVID11 | 70 | Female | Floor | 1.38 | - | 248 | Alive | NA |
| COVID12 | 52 | Male | ICU | 1.44 | 748 | 228 | Alive | 0 (Heparin) |
| COVID13 | 25 | Male | Floor | 31.16 | 128 | 156 | Alive | 0 (Enoxaparin) |
| COVID14 | 73 | Female | Floor | 0.95 | 860 | 294 | Alive | 3 (Enoxaparin) |
| COVID15 | 45 | Female | Floor | 0.99 | 514 | 378 | Alive | -1 (Enoxaparin) |
| COVID16 | 52 | Male | Floor | 0.89 | 506 | 236 | Alive | 1 (Enoxaparin) |
| COVID17 | 73 | Female | ICU | 0.24 | 437 | 173 | Deceased | 0 (Heparin) |
| COVID18 | 76 | Female | ICU | 10.48 | 765 | 481 | Deceased | 1 (Heparin) |
| COVID19 | 85 | Male | ICU | 0.77 | 701 | 264 | Alive | 0 (Enoxaparin) |
| COVID20 | 88 | Male | Floor | 5.82 | 462 | 56 | Alive | -3 (Enoxaparin) |
| COVID21 | 30 | Female | Floor | 0.59 | 506 | 212 | Alive | -1 (Enoxaparin) |
| COVID22 | 64 | Female | Floor | 0.75 | 449 | 155 | Deceased | -1 (Heparin) |
| COVID23 | 39 | Male | Floor | 0.48 | 498 | 194 | Alive | 1 (Enoxaparin) |
| COVID24 | 59 | Male | Floor | 0.19 | 773 | 187 | Alive | 6 (Enoxaparin) |
| COVID25 | 51 | Male | Floor | 0.82 | 755 | 205 | Alive | 1 (Enoxaparin) |
| COVID26 | 76 | Female | ICU | 10.48 | 765 | 481 | Deceased | 1 (Heparin) |
| COVID27 | 39 | Male | Floor | 0.7 | 660 | 200 | Alive | 0 (Enoxaparin) |
| COVID28 | 54 | Male | ICU | 1.44 | 673 | 277 | Alive | 0 (Enoxaparin) |
| COVID29 | 56 | Male | ICU | 21.35 | 559 | 267 | Deceased | 0 (Enoxaparin) |
| COVID30 | 56 | Male | Floor | 0.83 | 344 | 64 | Alive | 2 (Enoxaparin) |
| COVID31 | 75 | Female | ICU | 0.97 | 588 | 49 | Deceased | NA |
| COVID32 | 37 | Male | Floor | 0.35 | 553 | 369 | Alive | 1 (Enoxaparin) |
| COVID33 | 46 | Female | Floor | 0.5 | 321 | 187 | Alive | 1 (Enoxaparin) |
| COVID34 | 43 | Male | Floor | 0.62 | 462 | 281 | Alive | 0 (Enoxaparin) |
| COVID35 | 33 | Female | ICU | 0.84 | 587 | 230 | Alive | 0 (Heparin) |
| COVID36 | 56 | Male | Floor | 0.71 | 468 | 50 | Alive | 0 (Enoxaparin) |
| COVID37 | 60 | Female | Floor | 0.33 | 538 | 122 | Alive | 1 (Enoxaparin) |
| COVID38 | 62 | Female | ICU | 1.07 | 604 | 258 | Alive | 0 (Enoxaparin) |
| COVID39 | 65 | Male | Floor | 0.62 | 638 | 157 | Alive | NA |
| COVID40 | 42 | Male | Floor | 1.23 | 576 | 151 | Alive | 3 (Enoxaparin) |

#: Patient location at the time of enrollment; *: Plasma was collected on the day of enrollment for antibody testing. Positive numbers represent that the patient received heparin/Enoxaparin before plasma collection. Negative numbers represent that the patient received heparin/Enoxaparin after plasma collection. 0 represents that the patient received heparin/Enoxaparin on the day of plasma collection. NA represents that the patient did not receive heparin/Enoxaparin.

**Table S2. Information on the six RBD-binding antibodies cloned by Robbiani et al. that possess an RKH or Y_5_ motif and a κ-chain.**

| **Clone ID** | **HCDR3** | **VH gene** | **L-chain** |
| --- | --- | --- | --- |
| S1 | ARGNRLLYCSSTSCYLDAVRQG**YYYYYY**MDV* | VH1-69 | κ |
| S2 | ARDGAVVRFLEWPTVG**YYYYY**MDV | VH3-33 | κ |
| S3 | A**R**D**R**GG**H**DFWSGYGFYYYYGMDV | VH1-18 | κ |
| S4 | ANSPCSSASCKSG**YYYYY**MDV | VH3-23 | κ |
| S5 | VETNLWFGEDN**YYYYY**GMDV | VH3-23 | κ |
| S6 | A**R**VG**H**A**R**GVITGGDYFYYGMDV | VH1-18 | κ |

* = antibodies that shared the same VH but different VL. # = antibodies that shared the same VH and VL clonotypes. The RKH and Y_5_ motifs are shown with bold prints in red and and blue respectively.
